# Genotype-dependent responses to HIPV exposure in citrus: repression of *CsPUB21* and activation of SA/JA signaling

**DOI:** 10.3389/fpls.2025.1605151

**Published:** 2025-06-25

**Authors:** Raúl Ortells-Fabra, Carolina Gallego-Giraldo, Maria Angeles Forner-Giner, Alberto Urbaneja, Meritxell Pérez-Hedo

**Affiliations:** ^1^ Instituto Valenciano de Investigaciones Agrarias (IVIA), Centro de Protección Vegetal y Biotecnología, Unidad de Entomología, Moncada, Spain; ^2^ Instituto Valenciano de Investigaciones Agrarias (IVIA), Centro de Citricultura y Producción Vegetal, Moncada, Spain; ^3^ Instituto de Biología Molecular y Celular de Plantas (IBMCP), Consejo Superior de Investigaciones Científicas, Universitat Politècnica de València, Valencia, Spain

**Keywords:** jasmonic acid signaling, salicylic acid pathway, CsPUB21, volatile organic compounds, defense gene expression, biotic stress, abiotic stress, citrus rootstocks

## Abstract

Herbivore-induced plant volatiles (HIPVs) are known to activate immune signaling in plants; however, their effectiveness can vary depending on the genotype and the signaling pathway involved. In this study, we evaluated the transcriptional response of four citrus rootstocks (Carrizo citrange, Forner-Alcaide 5 (FA5), Forner-Alcaide 74 (FA74), and *Microcitrus australasica*) to six synthetic HIPVs [(Z)-3-hexen-1-ol, (Z)-3-hexenyl acetate, (Z)-3-hexenyl butyrate, (Z)-3-hexenyl propanoate, methyl jasmonate, and methyl salicylate]. We focused on genes associated with the salicylic acid (SA) and jasmonic acid (JA) pathways, as well as the susceptibility gene CsPUB21. Overall, the SA pathway was more consistently activated than the JA pathway, with upstream and intermediate genes induced across most genotypes and treatments. In contrast, downstream markers showed more variable expression, suggesting that synthetic HIPVs may induce a primed rather than fully activated defense state. Among the volatiles tested, (Z)-3-hexenyl propanoate and (Z)-3-hexen-1-ol were the most effective, activating genes in both pathways. Importantly, these two compounds also consistently repressed *CsPUB21* expression, a gene recently associated with huanglongbing (HLB) susceptibility, through coordinated transcriptional and post-translational regulation. Carrizo citrange showed the strongest transcriptional response, while FA74 exhibited more moderate activation, emphasizing the influence of genetic background on HIPV perception and signaling. These findings highlight the potential of selected synthetic HIPVs as sustainable defense priming agents capable of enhancing citrus immunity by simultaneously activating immune pathways and repressing susceptibility genes such as *CsPUB21*. This dual mode of action offers promising tools for the integrated management of HLB and other citrus diseases.

## 1 Introduction

Plants and herbivorous insects are engaged in a continuous evolutionary battle in which plants have evolved sophisticated defense strategies to mitigate herbivory (Kessler and Baldwin, 2002; Howe and Jander, 2008; War et al., 2012). Among these defenses, the production of herbivore-induced plant volatiles (HIPVs) and oviposition-induce plant volatiles (OIPVs) which represent reliable cues associated with presence of host eggs laid on plant tissues, a subset of volatile organic compounds (VOCs), plays a pivotal role in plant immunity (Dicke, 2009; Dicke and Baldwin, 2010). These airborne chemical signals serve dual functions in plant defense: they attract natural enemies of the herbivores (indirect defense) and act as inter-plant alarm signals. Upon detecting HIPVs, neighboring plants activate immune responses, particularly through the jasmonic acid (JA) and salicylic acid (SA) signaling pathways, which orchestrate the expression of defense genes (Turlings and Erb, 2018). This ability of plants to “eavesdrop” on the distress signals of their neighbors underscores the ecological significance of HIPVs in plant-insect interactions and provides a basis for innovative pest management strategies (Frost et al., 2008; Turlings and Erb, 2018).

Recent studies have demonstrated that the exogenous application of specific HIPVs can prime or induce defense responses in various plant species, thereby enhancing their resistance to pests and pathogens in an environmentally friendly manner. For instance, priming seeds with the indole volatile (emitted by maize during herbivory) boosted the resistance of *Arabidopsis thaliana* and *Medicago truncatula* plants against the beet armyworm *Spodoptera exigua* (Fab.) (Lepidoptera: Noctuidae) and the pea aphid *Acyrthosiphon pisum* (Harris) (Hemiptera: Aphididae), without compromising plant growth (Maurya et al., 2022). In maize, exposure to green leaf volatiles such as (Z)-3-hexenyl acetate is known to prime stronger anti-herbivore defenses upon subsequent attack (Engelberth et al., 2004). In tomato, foliar application of (Z)-3-hexen-1-ol was shown to induce both JA- and SA-mediated defenses directly, improving the plant’s resistance to the tobacco whitefly *Bemisia tabaci* (Gennadius) (Hemiptera: Aleyrodidae) while also increasing the emission of volatiles that attract whitefly parasitoids (Yang et al., 2020). Similarly, treating tomato plants with (Z)-3-hexenyl butyrate triggers early defense signaling events, including Ca_2_^+^ influx, activation of mitogen-activated protein kinases, and a rapid burst of reactive oxygen species, ultimately leading to defense responses such as stomatal closure (López-Gresa et al., 2018). Notably, the induction of these defenses by (Z)-3-hexenyl butyrate has been validated, resulting in enhanced resistance of crops to infections by the potato late blight disease, *Phytophthora infestans*, in potato and the rod-shaped, gram-negative bacterium *Pseudomonas syringae* in tomato (Payá et al., 2024).

Building on this concept, herbivore-induced volatiles are being explored as practical tools in agriculture. The use of slow-release dispensers emitting synthetic HIPVs has yielded promising results in pest control. For example, the green leaf volatile (Z)-3-hexenyl propanoate released in greenhouses significantly reduced infestations of key pests, such as the South American pinworm *Tuta absoluta* Meyrick (Lepidoptera: Gelechiidae) in tomato (Pérez-Hedo et al., 2021a) and the foxglove aphid *Aulacorthum solani* (Kaltenbach) (Hemiptera: Aphididae) in sweet pepper (Depalo et al., 2022). (Z)-3-hexenyl propanoate-exposed tomato crops induced overexpression of anti-herbivore defense genes, indicating activation of the jasmonate pathway and accumulation of defense metabolites in the plant (Pérez-Hedo et al., 2021a). Beyond annual crops, volatile defense elicitors have also been tested in perennial systems; for instance, exogenous application of methyl jasmonate (a naturally occurring VOC) in grapevine triggered the production of pathogenesis-related proteins and phytoalexins, resulting in increased resistance against powdery mildew in the vineyard (Belhadj et al., 2006). Taken together, these examples demonstrate the potential of synthetic HIPVs to enhance plant immunity and contribute to sustainable, integrated pest management, thereby reducing reliance on synthetic pesticides and their associated risks.

Citrus crops, which are of enormous global economic importance, are under intense pressure from a variety of pests and diseases that can severely impact yield and fruit quality (Urbaneja et al., 2020). Traditionally, citrus pest management has relied on chemical insecticides, a practice that provides short-term control but entails environmental and human health risks (Tudi et al., 2021). This has created an urgent need for sustainable alternatives. Inducing citrus defenses via HIPVs represents a novel approach to this challenge. To date, however, citrus plants have been largely overlooked in studies of VOC-induced resistance, possibly due to a historical focus on model plants (e.g., *Arabidopsis* and tomato) and the complexity of citrus biology, including its perennial growth and diverse genetics in rootstocks. Only recently has evidence emerged that citrus could respond to airborne defense cues. Recently, Pérez-Hedo et al. (2024a) demonstrated that exposing the rootstock Carrizo citrange [a hybrid of *Citrus sinensis* (L.) Osb. × *Poncirus trifoliata* (L.) Raf.] to (Z)-3-hexenyl propanoate activates their immune machinery. Exposed citrus plants showed strong upregulation of defense-related genes associated with both the SA and JA pathways, and this molecular response was manifested in reduced performance of the South African mealybug *Delottococcus aberiae* (De Lotto) (Hemiptera: Pseudococcidae) and the two-spotted mite *Tetranychus urticae* Koch (Acari: Tetranychidae) and increased attraction of those pests’ natural enemies (Pérez-Hedo et al., 2024a). This finding highlights the feasibility of enhancing citrus resistance through external volatile cues, opening the door to more ecologically based pest management strategies in orchards.

Despite this advance, it remains unknown how different synthetic HIPV compounds compare in their ability to induce defense responses in citrus, or whether such induction is consistent across different citrus genotypes. In the present study, we addressed this gap by investigating the effects of six HIPVs [(Z)-3-hexen-1-ol, (Z)-3-hexenyl acetate, (Z)-3-hexenyl butyrate, (Z)-3-hexenyl propanoate, methyl jasmonate, and methyl salicylate] on the expression of eight defense-related genes in four citrus rootstock species (Forner-Alcaide 5 (*C. reshni* Hort. Ex Tan. x *P. trifoliata*), Forner-Alcaide 74 (*C. reshni* x *P. trifoliata*), Carrizo citrange, and *Microcitrus australasica*). To capture the hierarchical structure of the defense signaling, we selected genes representing different functional levels within the SA and JA signaling pathways: two genes acting upstream in biosynthesis, one intermediate regulatory component, and one downstream marker of pathway activation. This approach enables us to evaluate whether synthetic HIPVs impact distinct stages of signaling and defense activation across different genotypes.

In addition, we incorporated the susceptibility gene *CsPUB21* into our analysis. This gene encodes a U-box E3 ubiquitin ligase involved in the degradation of the transcription factor MYC2, a central regulator of JA-mediated defense responses. Recent studies have shown that *CsPUB21* expression correlates positively with susceptibility to huanglongbing (HLB) (Zhao et al., 2025), the most devastating disease in citrus (Pérez-Hedo et al., 2025); and that its downregulation enhances MYC2 stability and resistance to infection. Given the known induction of MYC2 by synthetic HIPVs (Pérez-Hedo et al., 2024a), we explored whether volatile exposure could also affect *CsPUB21* expression. This could reveal whether HIPV-mediated priming activates defense signaling and also attenuates the expression of negative regulators, with potential implications for improving citrus resilience to HLB.

We hypothesized that exposure to these volatiles would differentially activate the citrus immune signaling network, specifically, the JA and/or SA defense pathways, as evidenced by enhanced transcription of genes across different functional levels. Understanding these dynamics may guide the identification of the most effective synthetic HIPVs and responsive citrus genotypes for use in stress management and resistance-oriented breeding strategies.

## 2 Materials and methods

### 2.1 Plant material

Four citrus rootstocks were selected for this study: Forner-Alcaide 5 (FA-5) and Forner-Alcaide 74 (FA-74), both hybrids of Cleopatra mandarin (*Citrus reshni*) and *Poncirus trifoliata*, developed at the Valencian Institute of Agricultural Research (IVIA); Carrizo citrange (CC), a hybrid of *Poncirus trifoliata* and *Citrus sinensis*, and *Microcitrus australasica* (F.Muell.) Swingle (Microcitrus) a hybrid of *Citrus reticulata* Blanco × *Microcitrus australis*. The rootstocks selected for this study were chosen based on their agronomic importance and relevance in citrus breeding, as well as their contrasting defence profiles. Carrizo Citrange is the most widely used rootstock in Spanish citrus production. FA5 has gained considerable popularity in recent years and is currently the most planted, while FA74 is expected to enter the market soon. Finally, *Microcitrus australasica* is increasingly being included in breeding programs due to its natural resistance to key diseases, including huanglongbing (HLB).

Seedlings were grown in 8 × 8 × 8 cm plastic pots filled with a mixture of 70% black peat and 30% perlite. All citrus plants were pesticide-free and watered twice a week. One of the waterings was supplemented with 2% citrus-specific fertilizer composed of NH_4_H_2_PO_4_ at 0.115 g/L, KNO_3_ at 0.065 g/L, Ca(NO_3_)_2_ at 1.25 g/L, and synthetic chelating at 0.018 g/L [Sequestrene^®^ (Syngenta NK 138Fe, Basel, Switzerland)] (Dahmane et al., 2022). Plants were maintained under controlled environmental conditions (25 ± 1°°C, 60% relative humidity, and a 14:10 h light:dark photoperiod). After approximately three months of growth, when they had developed 8 to 9 fully expanded leaves and reached a height of around 30 cm, the plants were considered physiologically suitable and selected for use in the bioassays.

### 2.2 Citrus plant’s exposure to synthetic GLVs

Six synthetic standards of volatile compounds were selected for this study based on their known roles in plant defence signaling: (Z)-3-hexen-1-ol (Z3C6OH), (Z)-3-hexenyl acetate (Z3-HA), (Z)-3-hexenyl butyrate (Z3-HB), (Z)-3-hexenyl propanoate (Z3-HP), methyl jasmonate (MeJA), and methyl salicylate (MeSA). All compounds were purchased from Sigma-Aldrich (St. Louis, MO, United States) with a reported purity of ≥98%. The (Z)-3-hexenyl derivatives were confirmed to contain >95% of the (Z)-isomer. Methyl jasmonate was used as a commercial mix of cis- and trans-isomers, and methyl salicylate was used in its standard, non-chiral form. All compounds were applied in pure (neat) form using low-density polyethylene (LDPE) diffusers as described below (Pérez-Hedo et al., 2021b; Riahi et al., 2022).

For each rootstock, an independent experiment was conducted under identical environmental conditions to evaluate the effect of six synthetic HIPVs and one mock control. Each treatment was assigned to a separate climate-controlled incubator (Sanyo MLR-350H, Sanyo, Japan), containing seven plants (biological replicates) of a single genotype. This design prevented cross-exposure between treatments and avoided inter-plant signaling effects. All incubators were run in parallel and maintained under the same conditions: 25 ± 1°C, 60% relative humidity, and a 14:10 h light:dark photoperiod. Each incubator was equipped with fluorescent lighting (15-watt white fluorescent tubes, FL15W, Sanyo Electric Co., Ltd.) positioned on the chamber ceiling. The light intensity at shelf level was approximately 160 µmol m²s¹ (photosynthetically active radiation, PAR), measured using a quantum light sensor. This setup ensured homogeneous light distribution across all trays and chambers. Each incubator was assigned to one of the seven treatments (six synthetic HIPVs and one mock), and seven individual plants of the corresponding rootstock were placed inside, serving as the biological replicates. Volatile application was achieved using low-density polyethylene (LDPE) polymer diffusers (Kartell, Fisher Scientific SL, Madrid, Spain) (Pérez-Hedo et al., 2021a), each containing 2 ml of the designated volatile compound in pure (neat) form and suspended within the incubator chamber. Plants were exposed continuously to the volatiles for 48 hours before sample collection.

### 2.3 RNA extraction and gene expression analysis

Apical tissue samples were collected from each plant 48 hours after exposure to synthetic HIPV. The samples were immediately flash-frozen in liquid nitrogen and ground to a fine powder for RNA extraction using NZYol reagent (NZYTech, Lisbon, Portugal). One microgram (µg) of total RNA from each sample was treated with the TURBO DNA-free™ Kit (Ambion^®^, Life Technologies, CA, USA) to eliminate any contaminating genomic DNA. Complementary DNA (cDNA) was synthesized by reverse transcription using the PrimeScript™ RT Reagent Kit (TAKARA Bio, CA, USA), following the manufacturer’s instructions.

Quantitative real-time PCR (qPCR) was performed using the LightCycler^®^ 480 System (Roche Molecular Systems, Inc., Switzerland) with NZYSpeedy qPCR Green Master Mix (2×) (NZYTech, Lisbon, Portugal), as described by Bouagga et al. (2018). Relative gene expression was calculated using the comparative Ct method (ΔΔCt) after validating primer efficiencies through the Relative Standard Curve Method (Applied Biosystems). The reference gene *CsGAPC1* (Glyceraldehyde-3-phosphate dehydrogenase, formerly *GAPDH*) was used for normalization. Primer sequences are listed in **Table 1**.

**Table 1:** List of genes and primers used in qPCR assays.

Gene	Gene name	Former Name	Locus	GenBank ID	Gene ID	Primer Sequence (5’→3’)	PCR PRODUCT (bp)
*CsGAPDH*	Glyceraldehyde-3-phosphate dehydrogenase	*CsGAPDH*	LOC102624117	XM_006476919	Cs5g06870	FW: GGAAGGTCAAGATCGGAATCAA RV: CGTCCCTCTGCAAGATGACTCT	75
*CsICS*	*Isochorismate synthase 2*, *Chloroplastic*	*CsICS*	LOC102630235	XM_006476588	Cs5g04210	FW: GGAGGAGGAGAGAGTGAATTTG	107
						RV: GGGTTGCTTCCTTCTACTATCC	
*CsPAL*	*Phenylalanine ammonia-lyase-like*	*CsPAL*	*LOC102620464*	XM_006481431	Cs6g11940	FW: CACATTCTTGGTAGCGCTTTG RV: AGCTACTTGGCTGACAGTATTC	94
*CsJAR1*	*Jasmonic acid-amino synthetase JAR1-like*	*CsJAR1*	LOC102611440	XM_006491176.4	orange1,1g007464m	FW: AAGGCGATGCAGTCACAATG	62
						RV: TGGTGGAAATCAGGACCAAAG	
*CsLOX2*	*Linoleate 13S-lipoxygenase 2-1, chloroplastic-like*	*CsLOX2*	LOC102629656	XR_001506736	orange1.1t03770	FW: GAACCATATTGCCACTTTCG	231
						RV: CGTCATCAATGACTTGACCA	
*CsCOI*	*Coronatine Insensitive 1*	*CsCOI*	LOC102620384	XM_006486308.3	Ciclev10031013m	FW: GGGAATGGAGGATGAAGAAGGT	61
						RV: GCCCTGAGCCAAAGCAATTA	
*CsMYC2*	*Transcription factor MYC2*	*CsMyc2*	LOC18049210	XM_024189896,1	orange1.1g046178m	FW: GGTGACCATGAGCTCCAACTG	171
						RV: GGCCGAAGAGAGATTTGGCTA	
*CsNPR1*	*Non-Pahogenesis Related Protein 1*	*CsNPR1*	LOC102617188	XM_006475416	Cs4g14600	FW: GTACCTTGAAAACAGAGTTGGACTGG	189
						RV: TGCTCCTCTTGCATTTTGAAAGGTG	
*CsPUB21*	*U-box domain-containing protein 21*	*CsPUB21*	At5g37490	XM_006469221	LOC102613708	FW: CGTTGGTCGTCGTCTATCGT RV: AAT GGA GAC TGC GAA CTC CG	150
*CsPR2*	*BTB/POZ domain and ankyrin repeat-containing protein NPR2 like*	*CsPR2*	LOC102617188	XR_371294.3	orange1,1g007849	FW: ACCTTAGACGAAGCCAATGCA	138
						RV: CAGACAACACCTTGGGATCACA	

To obtain a comprehensive overview of defense signaling, we selected eight genes that encompass different functional components of the SA and JA pathways. For the SA pathway, we evaluated *CsICS* (*Isochorismate synthase*) and *CsPAL* (*Phenylalanine ammonia-lyase*) as two upstream biosynthetic enzymes, *CsNPR1 (Non-pathogenesis-related protein 1)* as an intermediate regulatory gene, and *CsPR2* (*Pathogenesis-related protein 2*) as a downstream marker of SA pathway activation. For the JA pathway, we included two upstream genes*, CsLOX2* (*Lipoxygenase 2*) and *CsJAR1* (*Jasmonate resistant 1*), *CsCOI (Coronatine insensitive 1)* as an intermediate regulatory component, and *CsMYC2 (Transcription factor MYC2)* as a downstream effector. In addition, we included *CsPUB21*, a gene encoding a U-box E3 ubiquitin ligase recently identified as a negative regulator of JA signaling through MYC2 degradation (**Table 1**).

### 2.4 Statistical analysis

Results are presented as mean ± standard error (SE). Gene expression data were analyzed using one-way ANOVA, followed by Tukey’s *post hoc* test for mean separation at a significance level of P < 0.05. Statistical analyses were performed using GraphPad Prism version 10.4 (GraphPad Software, Boston, MA, USA). To generate heatmaps, data analysis was performed using R version 4.4.3. Gene expression data from pairwise combinations were transformed into a matrix format using the dcast function to calculate the mean expression values for each combination. Heatmaps were generated using the ComplexHeatmap package, employing a white-to-dark color gradient defined by the colorRamp2 function from the circlize package. Hierarchical clustering was performed on both rows and columns using Pearson correlation distance (1 - r) and complete linkage. The final visualization displayed annotated expression values within each cell.

## 3 Results

### 3.1 Expression of defense-related genes involved in the SA and JA pathways

#### 3.1.1 Gene expression in Carrizo citrange

**Figure 1:**
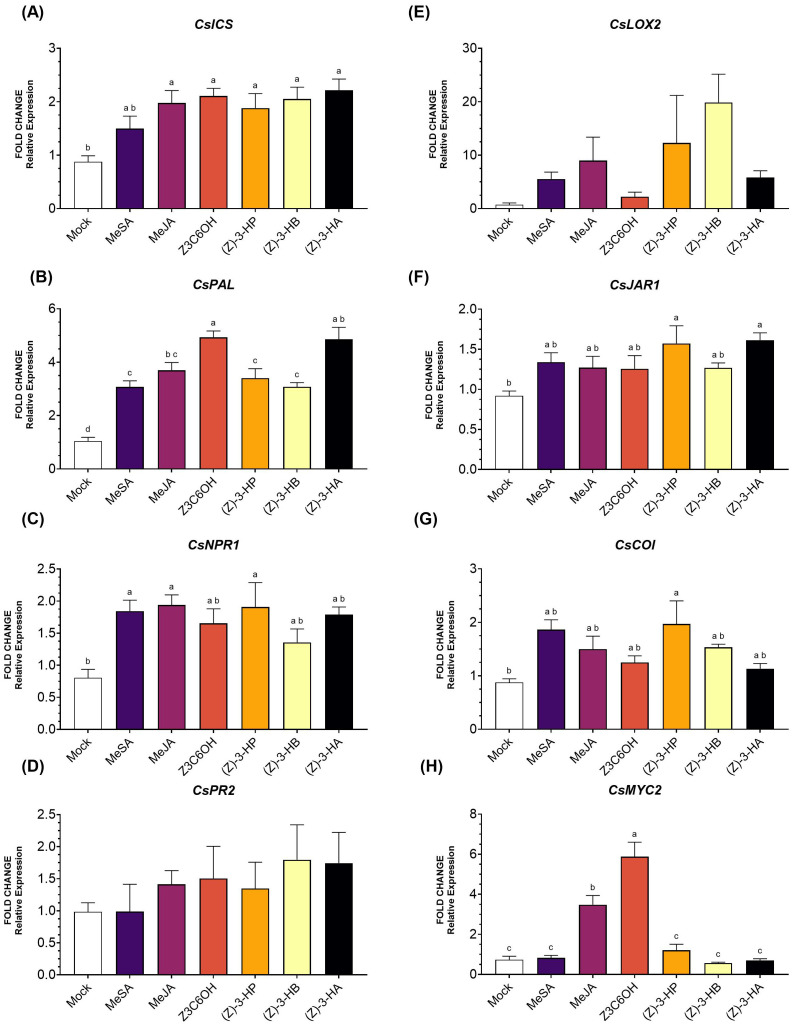
Expression of defense-related genes involved in the salicylic acid (SA) and jasmonic acid (JA) signaling pathways in Carrizo citrange rootstock exposed six synthetic HIPV treatments: methyl salicylate (MeSA), methyl jasmonate (MeJA), (Z)-3-hexenyl hexanoate (Z3C6OH), (Z)-3-hexenyl propanoate [(Z)-3-HP], (Z)-3-hexenyl butanoate [(Z)-3-HB], and (Z)-3-hexenyl acetate [(Z)-3-HA]. **(A)** *CsICS*, **(B)** *CsPAL*, **(C)** *CsNPR1*, **(D)** *CsPR2*, **(E)** *CsLOX2*, **(F)** *CsJAR1*, **(G)** *CsCOI*, **(H)** *CsMYC2*. Letters indicate significant differences between treatments based on Tukey’s test (*P* < 0.05).

Within the SA pathway, the upstream gene *CsICS* (**Figure 1A**) showed higher expression levels in all volatile treatments, except for MeSA (*F*_6,39_ = 5.077; *P* < 0.009). For the other upstream gene, *CsPAL* (**Figure 1B**), all treatments resulted in significantly higher expression (*F*_6,41_ = 25.92; *P* < 0.001), with Z3C6OH inducing the highest expression level. For the intermediate gene *CsNPR1* (**Figure 1C**), only MeSA, MeJA, and (Z)-3-HP induced significantly higher expression levels (*F*_6,41_ = 2.985; *P* = 0.018). Expression of the downstream marker *CsPR2* (**Figure 1D**) did not show significant differences in response to any of the volatile treatments (*F*_6,41_ = 0.583; *P* = 0.090).

In the JA pathway, *CsLOX2* expression (**Figure 1E**) was higher in all HIPV treatments; however, the high variability of the data resulted in no significant differences (*F*_6,28_ = 2.402; *P* = 0.061). Expression of *CsJAR1* (**Figure 1F**) was significantly increased only by (Z)-3-HP and (Z)-3-HA (*F*_6,40_ = 2.725; *P* = 0.028); other volatile caused moderate but non-significant increases. The expression of the intermediate marker gene *CsCOI* (**Figure 1G**) was only significantly induced by (Z)-3-HP (*F*_6,46_ = 2.973; *P* = 0.017). The transcription factor *MYC2* (**Figure 1H**) was strongly upregulated by Z3C6OH and MeJA, with Z3C6OH inducing the highest expression level and showing a significant difference from all other treatments (*F*_6,36_ = 30.44; *P* < 0.001). Expression levels for the remaining HIPVs were not significantly different from the mock.

#### 3.1.2 Gene expression in Forner Alcaide 5.

**Figure 2:**
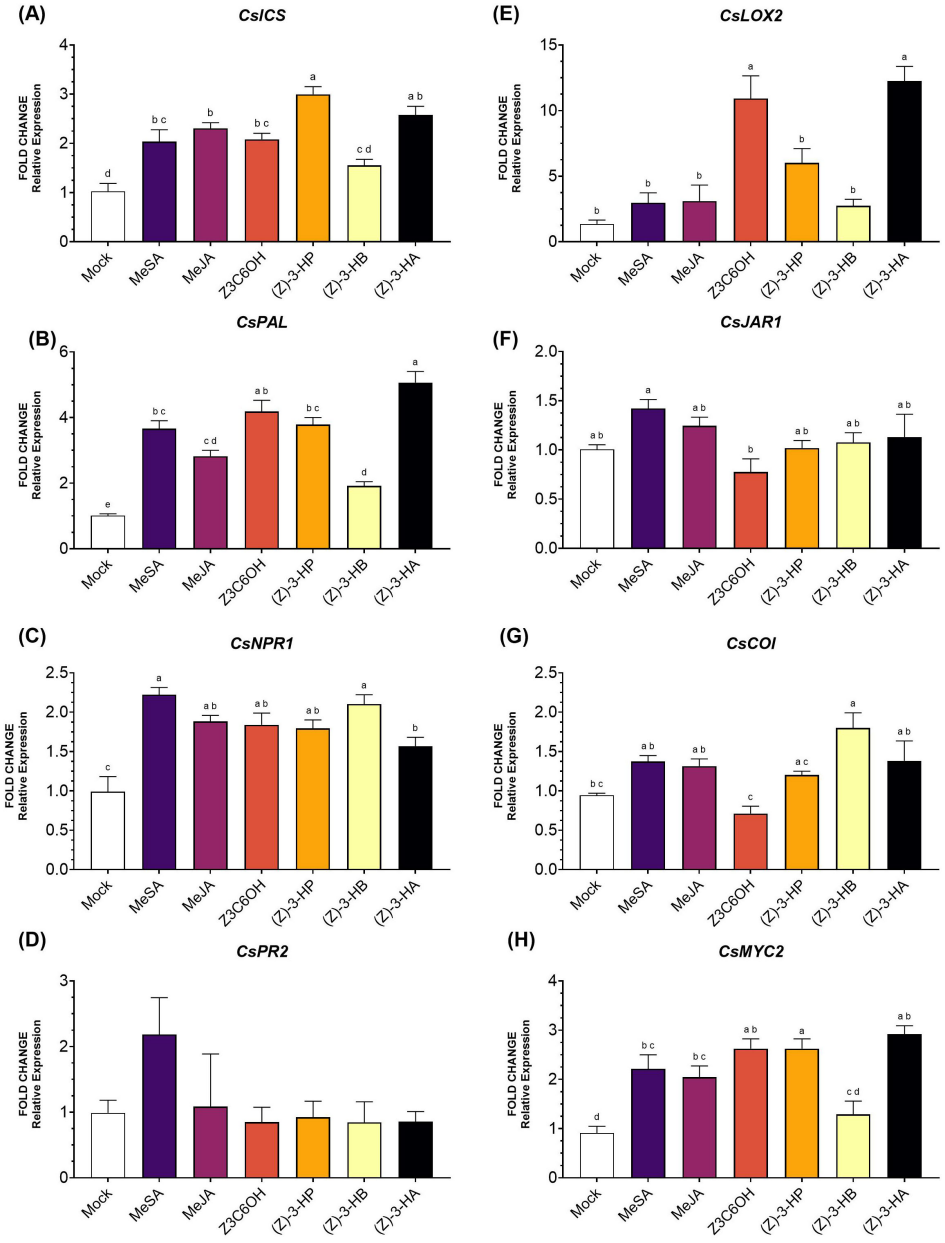
Expression of defense-related genes involved in the salicylic acid (SA) and jasmonic acid (JA) signaling pathways in Forner-Alcaide 5 (FA5) rootstock exposed six synthetic HIPV treatments: methyl salicylate (MeSA), methyl jasmonate (MeJA), (Z)-3-hexenyl hexanoate (Z3C6OH), (Z)-3-hexenyl propanoate [(Z)-3-HP], (Z)-3-hexenyl butanoate [(Z)-3-HB], and (Z)-3-hexenyl acetate [(Z)-3-HA]. **(A)** *CsICS*, **(B)** *CsPAL*, **(C)** *CsNPR1*, **(D)** *CsPR2*, **(E)** *CsLOX2*, **(F)** *CsJAR1*, **(G)** *CsCOI*, **(H)** *CsMYC2*. Letters indicate significant differences between treatments based on Tukey’s test (*P* < 0.05).

Within the SA pathway, the gene *CsICS* (**Figure 2A**) was significantly upregulated by MeSA, MeJA, Z3C6OH, (Z)-3-HP, and (Z)-3-HA (*F*_6,42_ = 16.54; *P* < 0.001). The strongest induction was observed for (Z)-3-HP, followed by (Z)-3-HA. The volatile (Z)-3-HB did not differ significantly from the mock. For the gene *CsPAL* (**Figure 2B**), expression was significantly higher in all treatments (*F*_6,38_ = 44.17; *P* < 0.001), with Z3C6OH showing the highest induction, followed by (Z)-3-HA. For the gene *CsNPR1* (**Figure 2C**), expression was significantly higher only in the MeSA, MeJA, and (Z)-3-HP treatments (*F*_6,47_ = 10.08; *P* < 0.001). Although some variation was observed, the expression of the *Cs*PR2 did not show significant differences in response to volatile treatments (**Figure 2D**) (*F*_6,44_ = 1.289; *P* = 0.285).

In the JA pathway, *CsLOX2* expression (**Figure 2E**) was induced by all HIPV treatments, but only Z3C6OH and (Z)-3-HA induced a statistically significant increase (*F*_6,35_ = 16.79; *P* < 0.001). The expression of *CsJAR1* (**Figure 2F**) was not significantly upregulated by any volatile treatment (*F*_6,44_ = 3.523; *P* = 0.006). Interestingly, *CsJAR1* expression was reduced after Z3C6OH exposure, although this difference was not statistically significant. However, it was significantly lower than the expression observed following MeSA exposure. The expression of *CsCOI* (**Figure 2G**) was only significantly induced by (Z)-3-HB (*F*_6,46_ = 6.164; *P* < 0.001). Interestingly, the expression level under Z3C6OH treatment, which was not significantly different from the mock treatment, was also lower than that observed for other volatiles such as MeSA, MeJA, (Z)-3-HB, and (Z)-3-HA. Finally, *CsMYC2* (**Figure 2H**) was significantly upregulated by all synthetic GLVs except (Z)-3-HB, with the strongest induction observed in response to (Z)-3-HP (*F*_6,41_ = 16.16; *P* < 0.001).

#### 3.1.3 Gene expression in Forner Alcaide 74.

**Figure 3:**
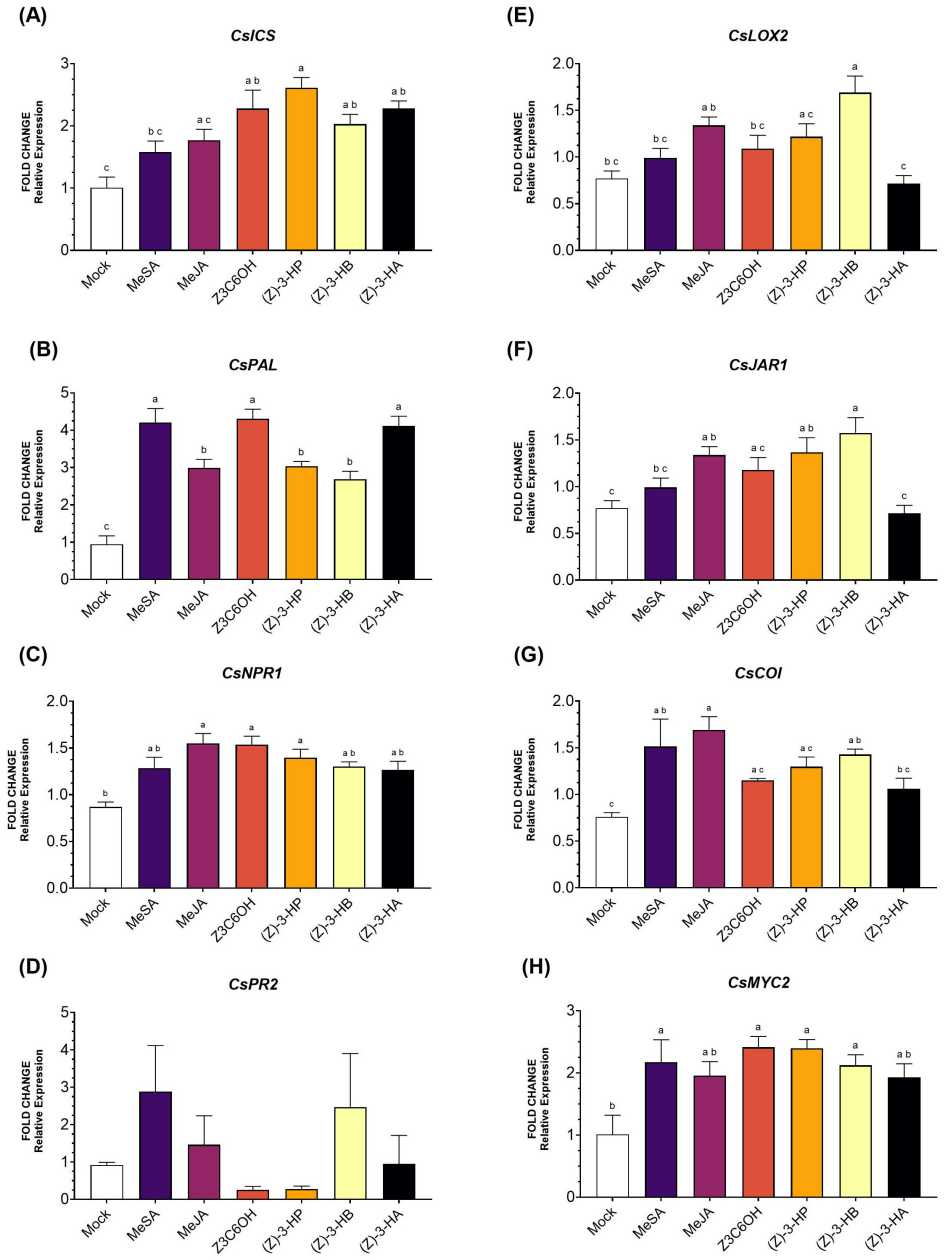
Expression of defense-related genes involved in the salicylic acid (SA) and jasmonic acid (JA) signaling pathways in Forner-Alcaide 74 (FA74) rootstock exposed six synthetic HIPV treatments: methyl salicylate (MeSA), methyl jasmonate (MeJA), (Z)-3-hexenyl hexanoate (Z3C6OH), (Z)-3-hexenyl propanoate [(Z)-3-HP], (Z)-3-hexenyl butanoate [(Z)-3-HB], and (Z)-3-hexenyl acetate [(Z)-3-HA]. **(A)** *CsICS*, **(B)** *CsPAL*, **(C)** *CsNPR1*, **(D)** *CsPR2*, **(E)** *CsLOX2*, **(F)** *CsJAR1*, **(G)** *CsCOI*, **(H)** *CsMYC2*. Letters indicate significant differences between treatments based on Tukey’s test (*P* < 0.05).

Within the SA pathway, *CsICS* (**Figure 3A**) was significantly upregulated by Z3C6OH, (Z)-3-HP, (Z)-3-HB, and (Z)-3-HA (*F*_6,44_ = 7.604; *P* < 0.001). Among these, (Z)-3-HP induced the highest expression levels. MeSA and MeJA also increased *CsICS* expression, although the differences were not statistically significant. For the gene *CsPAL* (**Figure 3B**), all synthetic GLVs treatments significantly increased expression, with MeSA, Z3C6OH, and (Z)-3-HA showing the strongest inductions (*F*_6,40_ = 22.06; *P* < 0.001). *CsNPR1* expression (**Figure 3C**) increased significantly after exposure to MeJA, Z3C6OH, and (Z)-3-HP (*F*_6,40_ = 4.734; *P* = 0.001). Despite some changes in expression levels of *CsPR2* (**Figure 3D**), the high variability observed between replicates resulted in no statistically significant differences across HIPV treatments (*F*_6,40_ = 1.600; *P* = 0.177).

In the JA pathway, *CsLOX2* expression (**Figure 3E**) was significantly upregulated only by (Z)-3-HB (*F*_6,41_ = 7.110; *P* < 0.001). Although other volatiles- except for (Z)-3-HA- also triggered higher expression than the control, the differences were not statistically significant. Similarly, *CsJAR1* expression (**Figure 3F**) was significantly increased in response to MeJA, (Z)-3-HP, and (Z)-3-HB treatments (*F*_6,37_ = 7.488; *P* < 0.001). In the case of *CsCOI* (**Figure 3G**), exposure to MeSA, MeJA, and (Z)-3-HB led to its overexpression (*F*_6,37_ = 4.661; *P* = 0.002). Finally, all volatile upregulated *CsMYC2* (**Figure 3H**), but only MeSA, Z3C6OH, (Z)-3-HP, and (Z)-3-HB showed significantly higher expression (*F*_6,39_ = 4.145; *P* = 0.003).

#### 3.1.4 Gene expression in microcitrus

**Figure 4:**
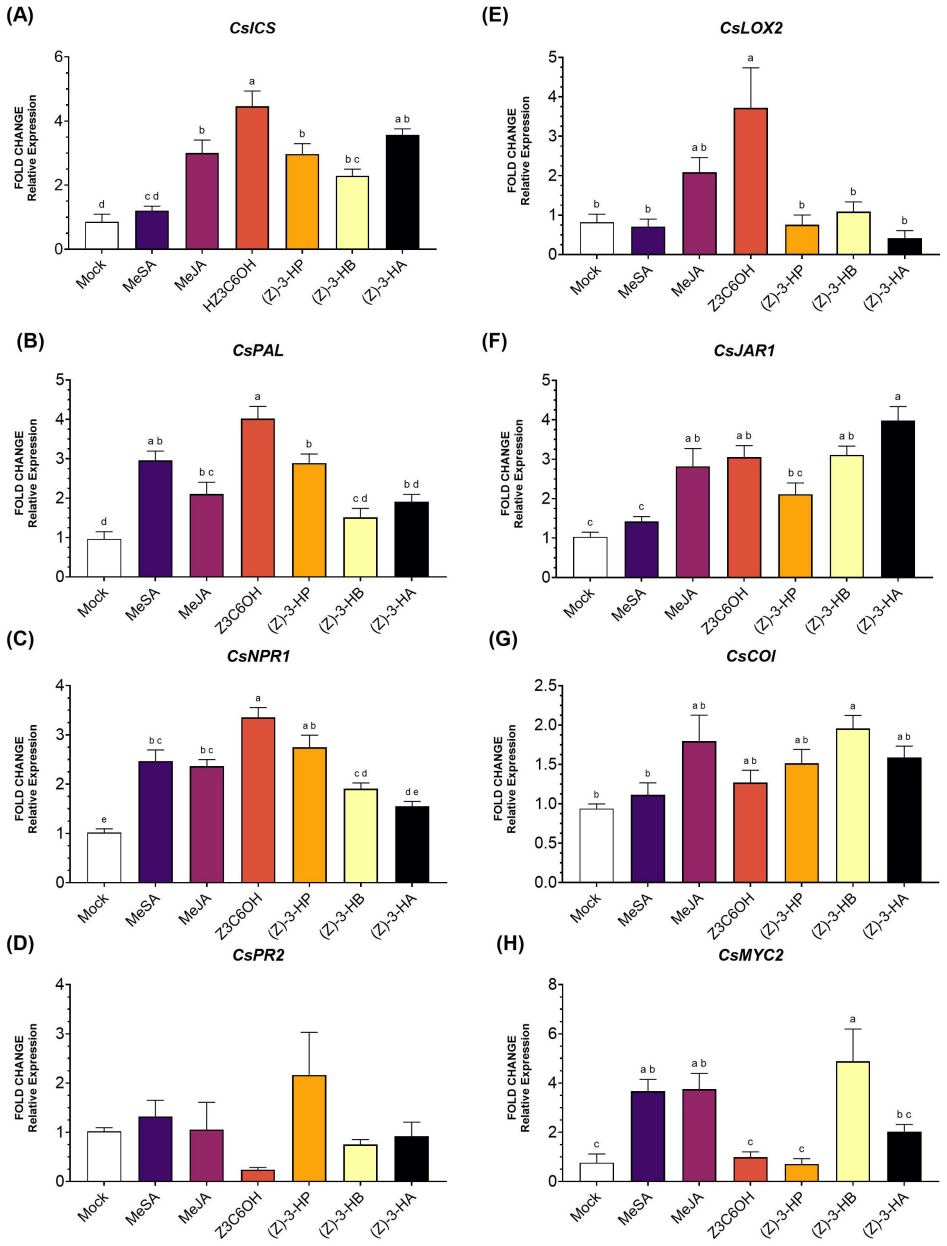
Expression of defense-related genes involved in the salicylic acid (SA) and jasmonic acid (JA) signaling pathways in Microcitrus rootstock exposed to six synthetic HIPV treatments: methyl salicylate (MeSA), methyl jasmonate (MeJA), (Z)-3-hexenyl hexanoate (Z3C6OH), (Z)-3-hexenyl propanoate [(Z)-3-HP], (Z)-3-hexenyl butanoate [(Z)-3-HB], and (Z)-3-hexenyl acetate [(Z)-3-HA]. **(A)** *CsICS*, **(B)** *CsPAL*, **(C)** *CsNPR1*, **(D)** *CsPR2*, **(E)** *CsLOX2*, **(F)** *CsJAR1*, **(G)** *CsCOI*, **(H)** *CsMYC2*. Letters indicate significant differences between treatments based on Tukey’s test (*P* < 0.05).

Within the SA pathway, the gene *CsICS* (**Figure 4A**) was significantly upregulated by all HIPV treatments, except for MeSA (*F*_6,36_ = 19.18; *P* < 0.001). Z3C6OH triggered the highest expression levels. For the gene *CsPAL* (**Figure 4B**), expression was significantly increased by MeSA, Z3C6OH, and (Z)-3-HP, with Z3C6OH showing the strongest response (*F*_6,40_ = 17.64; *P* < 0.001). The remaining volatiles, MeJA and (Z)-3-HB, also resulted in higher expression levels than the mock, but the differences were not statistically significant. *CsNPR1* expression increased after exposure to all volatiles except (Z)-3-HA (*F*_6,43_ = 23.37; *P* < 0.001). As observed for the two upstream genes, Z3C6OH induced the highest activation, followed by (Z)-3-HP (**Figure 4C**). No statistically significant differences were observed in *CsPR2* expression between synthetic GLVs treatments and the mock control (*F*_6,46_ = 1.777; *P* = 0.128) (**Figure 4D**).

In the JA pathway, *CsLOX2* expression (**Figure 4E**) was significantly upregulated only by Z3C6OH (*F*_6,34_ = 7.699; *P* < 0.001). The expression of *CsJAR1* (**Figure 4F**) was significantly increased by MeJA, MeSA, (Z)-3-HB, and (Z)-3-HA (*F*_6,40_ = 15.90; *P* < 0.001). *CsCOI* (**Figure 4G**) was significantly upregulated only in response to (Z)-3-HB exposure (*F*_6,44_ = 3.511; *P* = 0.007). Finally, *CsMYC2* (**Figure 4H**) showed significantly higher expression only in response to MeSA, MeJA, and (Z)-3-HB. At the same time, the other volatiles did not differ significantly from the mock treatment (*F*_6,33_ = 9.146; *P* < 0.001).

### 3.2 Expression of the susceptibility-related gene *CsPUB21*

**Figure 5:**
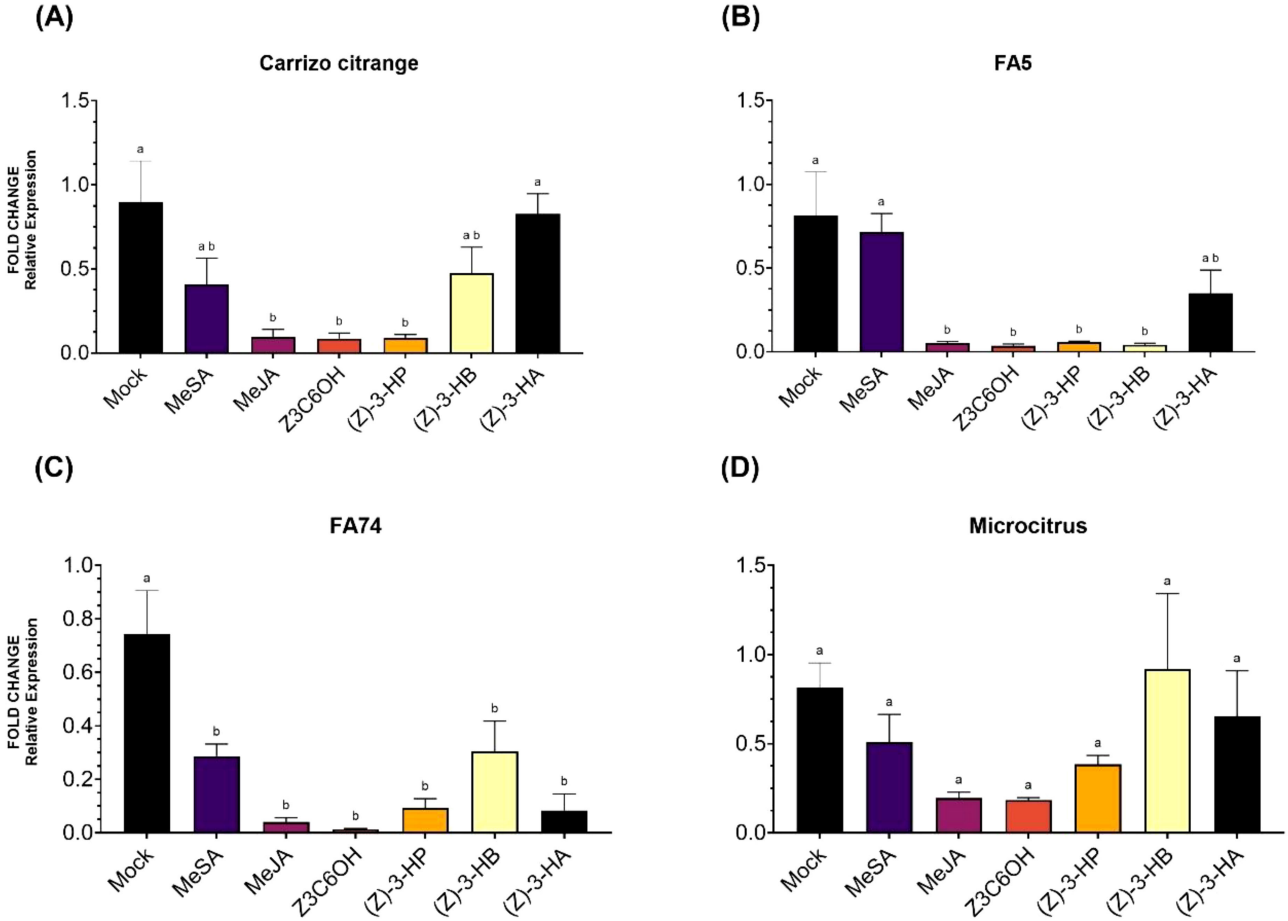
Relative expression of the susceptibility gene *CsPUB21* in citrus rootstocks (Carrizo citrange, FA5, FA74, and Microcitrus) exposed to six synthetic HIPV treatments: methyl salicylate (MeSA), methyl jasmonate (MeJA), (Z)-3-hexenyl hexanoate (Z3C6OH), (Z)-3-hexenyl propanoate [(Z)-3-HP], (Z)-3-hexenyl butanoate [(Z)-3-HB], and (Z)-3-hexenyl acetate [(Z)-3-HA]. **(A)** Carrizo citrange, **(B)** Forner-Alcaide 5 (FA5), **(C)** Forner-Alcaide 74 (FA74), **(D)** Microcitrus. Letters indicate significant differences between treatments based on Tukey’s test (*P* < 0.05).

Expression patterns of *CsPUB21* varied across citrus genotypes in response to HIPV treatments (**Figure 5**). In Carrizo citrange (**Figure 5A**), transcript levels were significantly reduced by MeJA, Z3C6OH, and (Z)-3-HP, which showed the lowest expression values among treatments (*F*_6_,_28_ = 6.695; *P* = 0.004). (Z)-3-HA maintained high expression levels comparable to the control, while MeSA and (Z)-3-HB resulted in intermediate values. In FA5 (**Figure 5B**), all treatments except MeSA and (Z)-3-HA significantly reduced *CsPUB21* expression compared to the mock (*F*_6_,_28_ = 8.213; *P* < 0.001), with the lowest levels observed for MeJA, Z3C6OH, (Z)-3-HP, and (Z)-3-HB. In FA74 (**Figure 5C**), all volatiles tested resulted in significant downregulation of CsPUB21 compared to the control (*F*_6_,_28_ = 9.884; *P* < 0.001), with Z3C6OH exhibiting the strongest repression. In contrast, in Microcitrus (**Figure 5D**), no significant differences were observed between treatments (*F*_6_,_28_ = 2.016; *P* = 0.097).

### 3.3 Gene expression heatmaps

**Figure 6:**
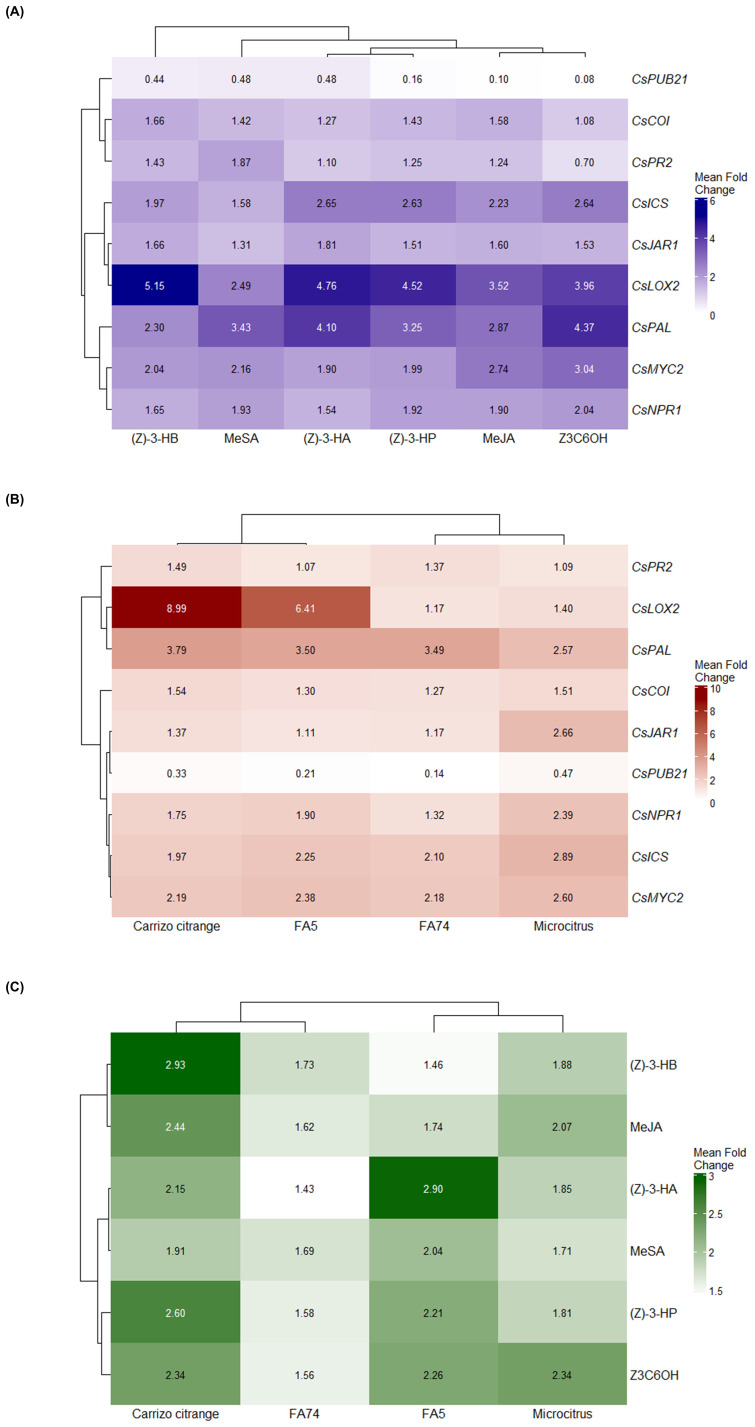
Heatmaps showing hierarchical clustering of fold-change expression levels of defense-related genes in citrus rootstocks exposed to synthetic HIPVs. Log_2_ fold-change (FC) values were calculated from qPCR data for eight defense-related genes (*CsICS*, *CsPAL*, *CsNPR1*, *CsPR2*, *CsLOX2*, *CsJAR1*, *CsCOI*, *CsMYC2*, and *CsPUB21*) following exposure to synthetic HIPVs. **(A)** Gene expression profiles under six synthetic HIPV treatments: methyl salicylate (MeSA), methyl jasmonate (MeJA), (Z)-3-hexenyl hexanoate (Z3C6OH), (Z)-3-hexenyl propanoate [(Z)-3-HP], (Z)-3-hexenyl butanoate [(Z)-3-HB], and (Z)-3-hexenyl acetate [(Z)-3-HA]. **(B)** Expression patterns across four citrus genotypes: Carrizo citrange, FA5, FA74, and Microcitrus. **(C)** Combined heatmap showing average gene expression responses across both synthetic HIPV treatments and genotypes. Hierarchical clustering was performed using Euclidean distance and complete linkage. Color scales represent log_2_ FC values, with darker shades indicating stronger induction levels.

**Figure 6** illustrates the transcriptional responses of defense-related genes under different volatile treatments and across citrus genotypes. The clustering analysis in **Figure 6A** reveals that the Z3C6OH and MeJA groups cluster together, as do the (Z)-3-HP and (Z)-3-HA groups, indicating that these volatiles elicit similar gene expression patterns. In contrast, MeSA and (Z)-3-HB form independent branches, suggesting distinct transcriptional profiles compared to the other HIPVs.

In **Figure 6B**, the hierarchical clustering of genotypes reveals that Carrizo and FA5 cluster together, indicating comparable and elevated transcriptional responses to volatile exposure. In contrast, FA74 and Microcitrus form a separate cluster, with Microcitrus exhibiting higher overall induction levels than FA74.

**Figure 6C** further displays the clustering of both genotypes and volatiles based on average gene expression levels. Among the volatiles, (Z)-3-HP and Z3C6OH form one cluster, while MeSA and (Z)-3-HA form another, indicating shared activation profiles within each pair. MeJA and (Z)-3-HB appear as separate branches, suggesting unique patterns of gene induction. These results demonstrate both compound- and genotype-dependent variation in transcriptional responses to synthetic HIPVs.

## 4 Discussion

Our results demonstrate that synthetic GLVs can activate immune signaling pathways in citrus rootstocks, with responses depending on both the hormone pathway and the genotype. Notably, we observed the downregulation of *CsPUB21*, a key susceptibility gene linked to MYC2 regulation and HLB sensitivity.

Exposure to synthetic HIPVs generally triggered the activation of upstream and intermediate genes in the SA pathway, including *CsICS, CsPAL*, and *CsNPR1*, as indicated by their fold-change values in **Figure 6A**. In contrast, *CsPR2* displayed much lower and more variable expression, suggesting that volatile exposure may selectively enhance early steps of the signaling cascade without fully activating downstream defense effectors. This pattern supports the hypothesis that these volatiles induce a primed state, in which plants are metabolically prepared to respond more rapidly and effectively to subsequent stress (Engelberth et al., 2004; Ton et al., 2006; Frost et al., 2008; Erb et al., 2015). However, this interpretation remains hypothetical and would need to be validated through challenge experiments involving actual biotic stress to confirm that the plants are indeed primed for enhanced defence.

In addition to priming, other regulatory mechanisms may explain the limited activation of downstream defense genes such as *CsPR2*. Antagonistic hormonal crosstalk, particularly from JA or ABA, could interfere with SA signaling or limit the activity of key regulators such as *NPR1*. Notably, *NPR1* requires interaction with TGA transcription factors to activate SA-responsive genes, and their availability or activity may be constrained under certain hormonal conditions (Zhang et al., 1999). These multilayered controls, including feedback loops and energy-saving strategies, help fine-tune immune responses, allowing the plant to balance growth and defense depending on environmental cues (Huot et al., 2014; Zhou et al., 2015).

When examining the JA-related genes in the heatmap (**Figure 6**), *CsLOX2* and *CsMYC2* stand out as the most strongly induced genes within the JA signaling group. *CsLOX2*, which participates in the initial steps of JA biosynthesis, showed consistent upregulation across treatments, particularly under (Z)-3-HA and (Z)-3-HP. However, this induction does not necessarily imply full activation of the jasmonic acid pathway, as reflected by the low expression levels of intermediate genes such as *CsJAR1* and *CsCOI*. It is possible that *CsLOX2* is acting independently, or that its activation does not culminate in the production of bioactive JA derivatives, since LOX genes can participate in various oxylipin-related processes. *CsMYC2* also exhibited strong expression, especially in response to Z3C6OH and MeJA, suggesting that HIPVs may preferentially enhance late-stage components of the JA cascade. These findings support the hypothesis of transcriptional reprogramming centered on *MYC2*, independently of the full activation of the upstream JA biosynthetic machinery. As proposed by Song et al. (2022), *MYC2* functions as a central integrator of stress signals and can activate defense genes in response to specific stimuli, including HIPVs, even in the absence of complete JA biosynthesis. Such activation may represent a priming-like state, allowing the plant to prepare for future attack with minimal metabolic cost. Supporting this hypothesis, we found that the expression of *CsPUB21*, an E3 ubiquitin ligase responsible for *MYC2* degradation (Zhao et al., 2025), was consistently repressed across all volatile treatments and genotypes. Fold-change values remained below 0.5 in every case. This suggests that HIPVs may activate *MYC2* at the transcriptional level and reduce its proteolytic turnover, contributing to its stabilization. This dual regulatory effect, involving the activation of *MYC2* and the suppression of its negative regulator, adds a new layer of control to volatile-mediated defense priming in citrus.

Among the four genotypes evaluated, Carrizo and FA5 exhibited the most robust transcriptional responses to HIPV exposure, as observed in their clustering and expression levels in **Figure 6B**, with strong induction of genes in both the SA and JA pathways. This suggests a higher sensitivity to volatile cues and more efficient signal transduction in these rootstocks. In contrast, FA74 showed the weakest activation overall, particularly within the JA pathway, while Microcitrus displayed a broad but moderate activation pattern. These genotypic differences may have practical implications for selecting rootstocks that enhance pest and disease resistance under integrated management strategies.

The observed variability in gene activation among citrus rootstocks likely reflects their distinct genetic backgrounds, which influence volatile perception, basal defense status, and signaling capacity. The stronger response of Carrizo citrange and FA5 may be attributed to their *P. trifoliata* lineage, known for its role in conferring resistance to both biotic and abiotic stressors (Forner-Giner et al., 2020; Peng et al., 2020; Wang et al., 2024). Recent studies have shown that *P. trifoliata* contributes to enhanced expression of defense-related genes in response to synthetic HIPVs (Pérez-Hedo et al., 2024a). In parallel, species of the *Microcitrus* genus, such as *Microcitrus australasica*, are naturally resistant to a broad range of citrus pathogens, including CLas, the causal agent of HLB (Huang et al., 2021). This resistance has positioned *Microcitrus* as a valuable genetic resource for both conventional breeding and biotechnological approaches (Alquézar et al., 2021).

Interestingly, despite its strong resistance phenotype, *Microcitrus australasica* exhibited the highest *CsPUB21* expression among the four genotypes and showed no significant repression of this gene following volatile treatment. This suggests a fundamentally different regulatory context for *CsPUB21* in this species. According to Zhao et al. (2025), Microcitrus lacks a helitron insertion in the *CsPUB21* promoter that is present in susceptible species such as *C. sinensis*, which reduces its responsiveness to JA-mediated induction via *MYC2* binding sites. Additionally, although *Microcitrus* expresses low levels of the dominant-negative paralog *PUB21DN*, this mechanism may still partially mitigate *PUB21* activity. Together, these findings suggest that in Microcitrus, resistance may not depend on the transcriptional repression of *PUB21*, but instead on structural and functional divergence that diminishes its immunosuppressive role.

SA plays a central role in defense against biotrophic pathogens, and its importance in limiting *C*Las colonization and symptom progression is increasingly supported by experimental evidence (Liu et al., 2023; Pérez-Hedo et al., 2024b; Yang et al., 2024). Based on this, priming SA-related defenses prior to pathogen exposure emerges as a promising strategy to improve citrus tolerance to HLB. Our findings support this approach, showing that selected HIPVs can pre-activate components of both SA and JA pathways while simultaneously repressing *CsPUB21*, a gene associated with increased HLB susceptibility. This dual action may reinforce plant immunity and promote a sustained defense-ready state. Applied in nurseries or during early orchard establishment, synthetic HIPV treatments could serve as a proactive tool to strengthen the plant’s immune system before infection occurs, contributing to more effective and sustainable HLB management strategies. This is a critical question for future research, especially in the context of field applications where transient exposure or pulsed treatments may be more practical. Encouragingly, studies in other crops using a single, transient biotic stimulus, such as the brief exposure to herbivory by the predatory mirid *Nesidiocoris tenuis* Reuter (Hemiptera: Miridae), have shown that defence responses can persist for up to 14 days (Bouagga et al., 2018). Whether a similar persistence can be achieved with synthetic volatiles in citrus is currently unknown and will be addressed in future work aimed at characterizing the temporal window of the primed state.

Among the tested volatiles, (Z)-3-HP and Z3C6OH consistently stood out for their robust transcriptional impact and for promoting *CsPUB21* repression, a pattern confirmed by their grouping and high average expression values in **Figure 6C**. These dual effects, which enhance immune signaling and reduce the expression of a key susceptibility gene, position them as promising agents for defense priming. This aligns with our previous transcriptomic study with (Z)-3-HP, which showed broad transcriptional activation of genes related to plant immunity, stress tolerance, and redox balance in citrus (Pérez-Hedo et al., 2024a), reinforcing the potential of this compound for integrated pest and disease management.

Overall, our findings underscore the potential of (Z)-3-HP and Z3C6OH as robust priming agents to enhance citrus defense responses. Their consistent activation of SA and JA signaling, combined with the suppression of the susceptibility gene *CsPUB21*, suggests a coordinated mechanism that strengthens immunity at multiple regulatory levels. Given their broad-spectrum effects across genotypes, these synthetic HIPVs represent promising candidates for sustainable citrus protection (Farag et al., 2005; Yang et al., 2020; Pérez-Hedo et al., 2021b, 2024a). However, their practical implementation requires further validation under field conditions and in grafted plants, considering that rootstock-scion interactions may influence responsiveness.

Future work will focus on consolidating and expanding the validation of synthetic HIPVs under field conditions. We have been conducting field experiments for the past three years across three commercial citrus orchards in eastern Spain. These trials are evaluating the long-term effects of selected volatile compounds on disease resistance (including HLB), pest population dynamics, and agronomic performance. Treated trees are being compared with untreated controls to assess differences in disease incidence, physiological stress, and fruit yield. Building on these results, upcoming studies will explore the effectiveness of pulsed and transient exposure strategies, as well as rootstock–scion interactions that may influence responsiveness to synthetic HIPVs. This ongoing work aims to establish practical, low-input protocols for integrating HIPV-based priming into sustainable citrus production systems.

## Data Availability

The datasets presented in this study can be found in online repositories. The names of the repository/repositories and accession number(s) can be found in the article/supplementary material.
